# Oncogenomic disruptions in arsenic-induced carcinogenesis

**DOI:** 10.18632/oncotarget.15106

**Published:** 2017-02-05

**Authors:** Adam P. Sage, Brenda C. Minatel, Kevin W. Ng, Greg L. Stewart, Trevor J.B. Dummer, Wan L. Lam, Victor D Martinez

**Affiliations:** ^1^ Department of Integrative Oncology, British Columbia Cancer Research Centre, Vancouver, British Columbia, Canada; ^2^ Centre of Excellence in Cancer Prevention, School of Population and Public Health, University of British Columbia, Vancouver, British Columbia, Canada

**Keywords:** arsenic, cancer, genetics, epigenetics, non-coding RNA

## Abstract

Chronic exposure to arsenic affects more than 200 million people worldwide, and has been associated with many adverse health effects, including cancer in several organs. There is accumulating evidence that arsenic biotransformation, a step in the elimination of arsenic from the human body, can induce changes at a genetic and epigenetic level, leading to carcinogenesis. At the genetic level, arsenic interferes with key cellular processes such as DNA damage-repair and chromosomal structure, leading to genomic instability. At the epigenetic level, arsenic places a high demand on the cellular methyl pool, leading to global hypomethylation and hypermethylation of specific gene promoters. These arsenic-associated DNA alterations result in the deregulation of both oncogenic and tumour-suppressive genes. Furthermore, recent reports have implicated aberrant expression of non-coding RNAs and the consequential disruption of signaling pathways in the context of arsenic-induced carcinogenesis. This article provides an overview of the oncogenomic anomalies associated with arsenic exposure and conveys the importance of non-coding RNAs in the arsenic-induced carcinogenic process.

## INTRODUCTION

**Figure 1:**
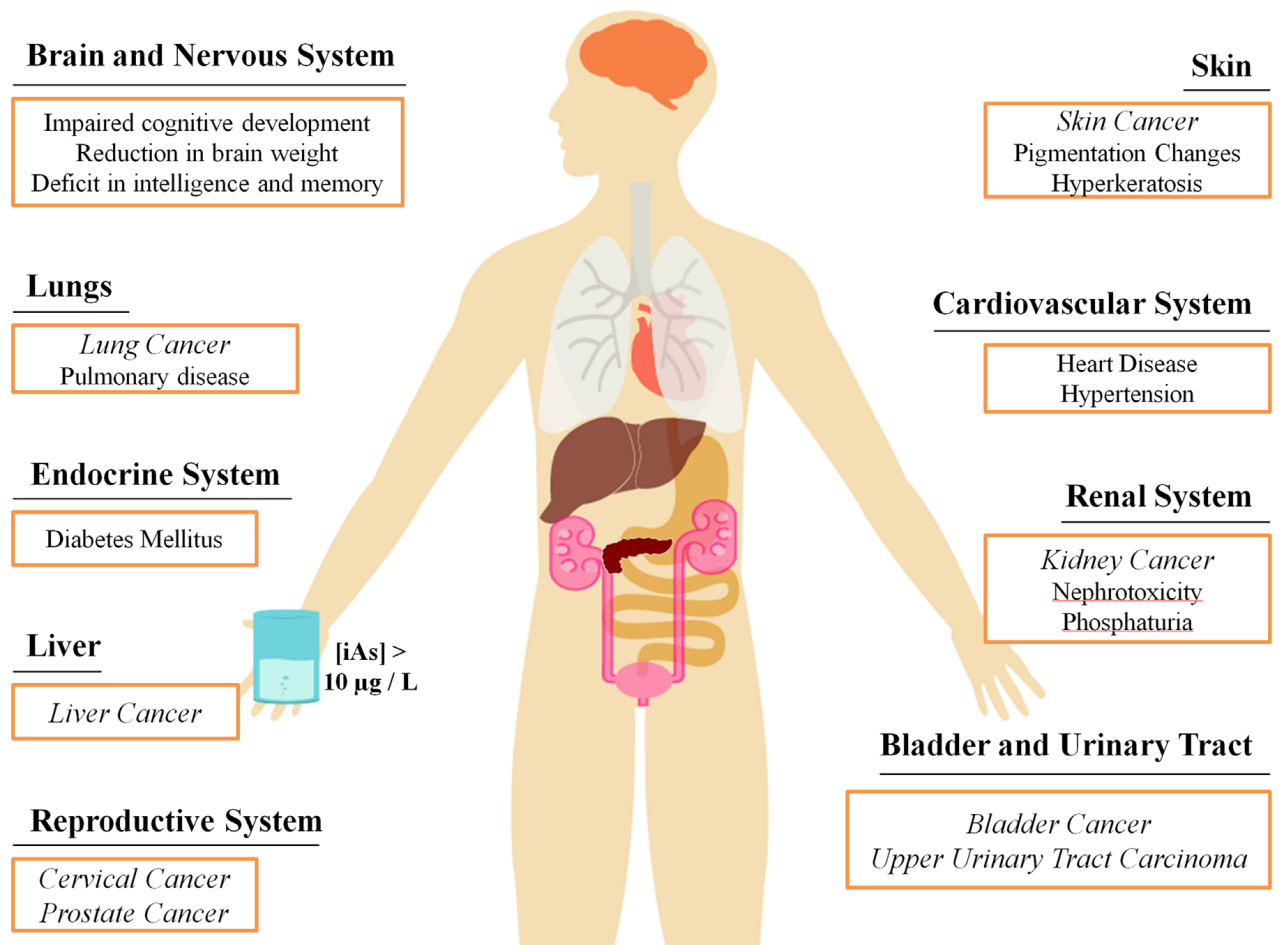
Health effects associated with chronic exposure to inorganic arsenic from contaminated drinking water Levels of iAs in drinking water near the maximum threshold of 10μg/L can lead to the onset of many diseases in a number of areas in the body. Cancer is a particularly prevalent disease resulting from chronic arsenic exposure, represented in italics.

**Figure 2:**
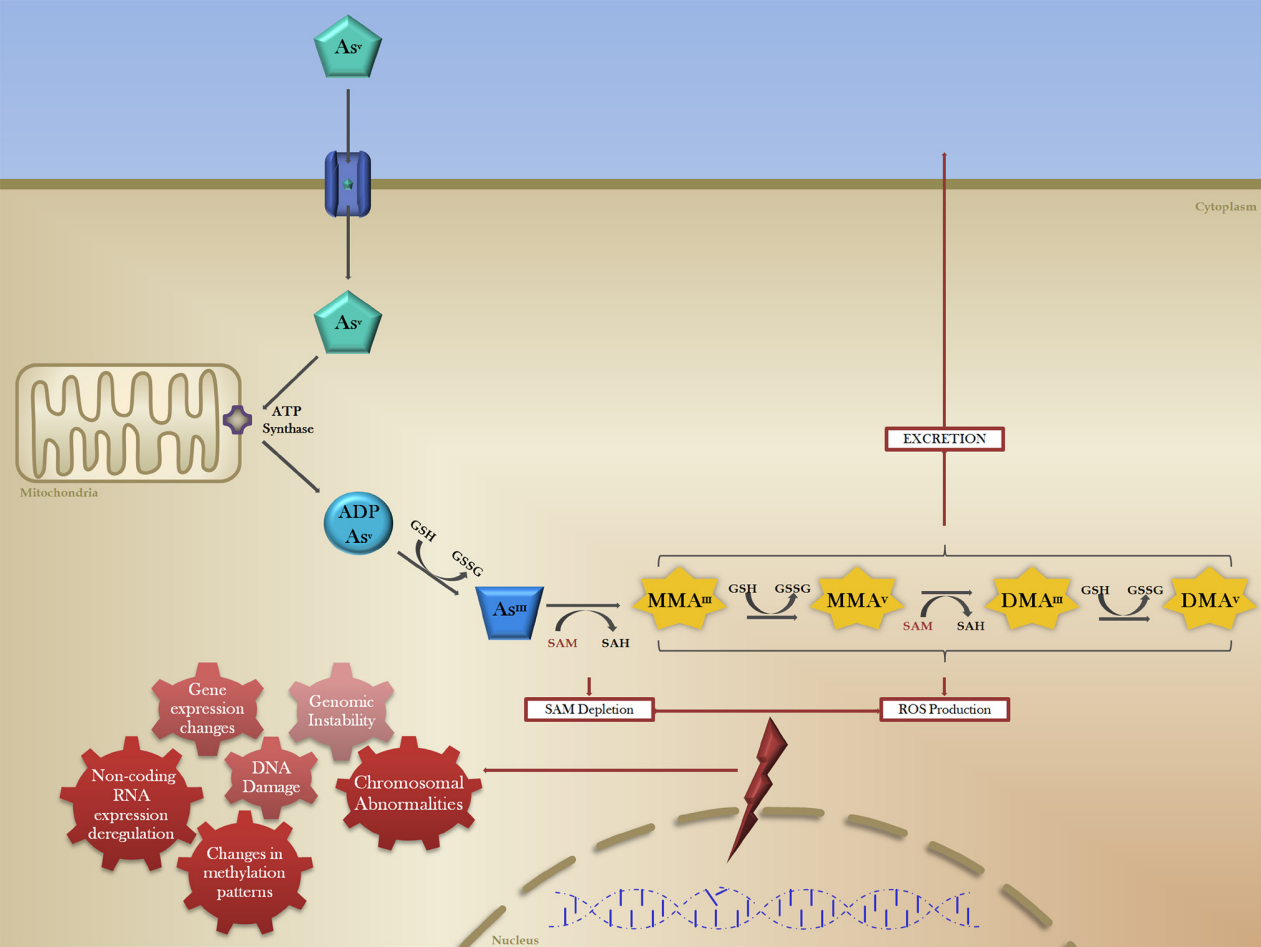
The biotransformation of inorganic arsenic and mechanisms of arsenic-induced carcinogenesis The reduction, oxidation and methylation of pentavalent arsenic (As^V^, green pentagon) occurs after cellular intake via membrane transport proteins (blue cylinder). Mitochondrial ATP synthase (purple) conjugates As^V^ with ADP, which is then reduced by the electron donor glutathione (GSH) to produce As^III^ (blue trapezoid), a more cytotoxic form of arsenic. In order for excretion, As^III^ is methylated with methyl groups donated by S-adenosylmethionine methyltransferase (SAM). These methylated arsenic species (MMA, DMA; yellow) all have carcinogenic potential through the induction (red lightning bolt) of a number of genomic and epigenetic effects (red gears), culminating in transcriptomic changes and generalized genomic instability.

Arsenic is an environmental carcinogen associated with human skin, bladder, liver and lung cancers [1, 2]. According to the World Health Organization (WHO), 10 μg/L is the maximum acceptable arsenic concentration in drinking water, however, high levels of arsenic have been found in groundwater in more than 70 countries across 5 continents, including North America, affecting over 200 million people [3–7]. Environmental arsenic in groundwater is predominantly found in the inorganic form (iAs), as pentavalent arsenate (As^V^) [8]. The consequences of chronic exposure to low doses lead to deleterious effects in multiple organs and tissues (Figure 1). The oncogenic effect is in part attributed to the production of toxic metabolites in the biotransformation of arsenic (Figure 2).

Among the symptoms of chronic exposure to iAs are changes in skin pigmentation, hyperkeratosis (abnormal thickening of the skin) and other skin lesions [9]. These lesions may be precursors to several types of skin cancer, which is the most prevalent form of arsenic-induced cancer [10, 11]. In addition, iAs exposure also appears to play a role in the development of bladder, liver and lung cancers [12–15] though evidence also points to an increased risk for other tissue types, such as breast, prostate and cervix [14, 16–18]. More recent evidence suggests an increased risk of urinary tract cancer with exposure to arsenic in drinking water at around guideline levels (i.e. 10 μg/L) [19–22]. Furthermore, iAs is reported to be associated with pulmonary disease, cardiovascular diseases, and neurodevelopmental and cognitive impairments, which can even be observed in newborns of mothers previously exposed to arsenic [23–29] (Figure 1).

The occurrence of different organ-specific malignancies associated with arsenic exposure may be a consequence of its transit and storage functions, namely its routes of entry to the human body (e.g. inhalation, adsorption and ingestion), as well as its metabolism and excretion, the latter being correlated with the higher incidence of kidney, urinary tract and bladder cancers [21–22]. Metabolically, cellular intake of As^V^ occurs through membrane transport proteins including aquaporins and inorganic phosphate (Pi) transporters (Figure 2) [30, 31]. Mitochondrial ATP synthase, which is able to use As^V^ instead of Pi to produce ATP, conjugates ADP with As^V^, which is then reduced to the more cytotoxic As^III^ by glutathione (GSH) [32]. The high toxicity of As^III^ is partly the result of its strong interaction with protein thiol groups, which can trigger inactivation and proteolysis of key tumour-suppressor proteins [33].

Arsenic toxicity is dependent on multiple factors. Molecular alterations at the DNA and RNA level may be at the forefront of this issue, including the disruption of DNA damage-repair mechanisms, coding and non-coding gene expression alterations and changes in mutation patterns [34–36]. This is further complicated by individual factors, such as genetic polymorphisms that may disrupt the intake-excretion balance [37], which may regulate the susceptibility to arsenic-induced damage, as well as lifestyle, which may make individuals with obesity more efficient in the methylation and excretion of arsenic [38]. Interestingly, arsenic trioxide (As_2_O_3_) displays anti-tumour activity and as such is currently used as a chemotherapeutic agent in the treatment of acute promyelocytic leukemia (APL), particularly in cases with a translocation between chromosomes 15 and 17 [39]. As_2_O_3_ is associated with a number of genetic and epigenetic changes, including alterations in coding and non-coding gene expression levels and abnormal methylation patterns [40, 41]. In light of this, it is important to examine the molecular changes in both the treatment of APL with As_2_O_3_ in addition to iAs exposure to fully understand the mechanisms of arsenic-induced carcinogenesis.

**Figure 3:**
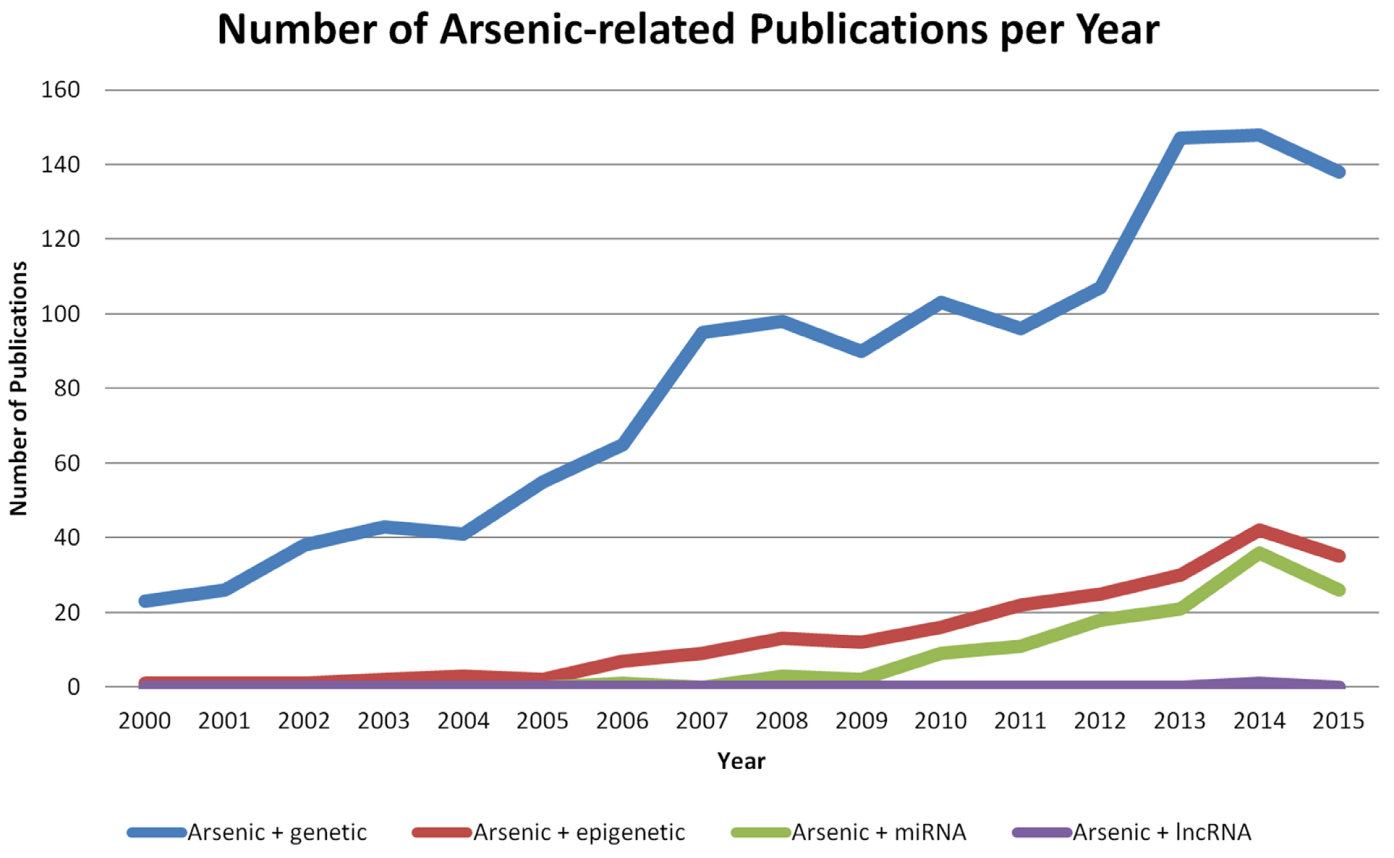
Number of publications relating genetics and epigenetics to arsenic exposure Search was performed within EndNote (Version 7, Thomson Reuters) and manually filtered. Number of publications are based on a United States National Library of Medicine PubMed search using the terms “arsenic AND genetic” (blue line), “arsenic AND epigenetic” (red line), “arsenic AND miRNA OR microRNA” (green line), or “arsenic AND lncRNA OR lincRNA OR long non-coding RNA” (purple line). 2016 publications were not included in the search, and annual (Jan 1-Dec 31) date limitations were used.

As literature relating (epi)genetics to arsenic exposure has been accumulating at an increasing rate (Figure 3), we review the latest advances in the oncogenomic effects of arsenic-induced carcinogenesis.

## GENOMIC ABERRATIONS ASSOCIATED WITH ARSENIC

### Oxidative DNA damage

#### Carcinogenic aspects of arsenic exposure

Several studies propose that iAs is not able to bind directly to DNA and therefore is not likely to be responsible for mutational damage [42]. However, methylated arsenicals derived from iAs biotransformation have been shown to generate single and double-stranded DNA breaks through the formation of reactive oxygen species (ROS) [43]. Human keratinocytes exposed to arsenic produce two main types of ROS: superoxide anion (O_2_^−^ ·) and hydrogen peroxide (H_2_O_2_) [44]. The type of ROS produced in response to arsenic is cell-type specific, highlighting the relevance of this mechanism to arsenic-induced carcinogenesis [45, 46]. In fact, the mechanism of oxidative stress is the most studied features of arsenic toxicity and is recognized as one of the most important [47].

The basis of the carcinogenic aspects of oxidative stress upon exposure to arsenic is that when attacking DNA, ROS produce 8-Hydroxy-29-deoxyguanosine (8-OHdG), which is capable of generating G>T conversions that trigger G>C → T>A transversions [48–50]. 8-OHdG is a biomarker of DNA oxidative damage, shown to be expressed at higher levels in the epidermal nuclei of arsenic-related Bowen's disease, Bowen's carcinoma and arsenic keratosis [51–53]. Furthermore, whole-genome sequencing analysis revealed a specific mutational signature that can differentiate arsenic-related lung tumours from tumours unrelated to arsenic, even though they may display the same histological features. Arsenic-related tumours are characterized by low overall number of mutations, high rates of T>G/A>C, and low rates of C>A/G>T transversions [34].

Arsenic-induced mutations can be particularly damaging if they lead to the activation of an oncogene, such as *RAS* [54]. Mice exposed to arsenic during gestation have higher incidence of liver tumours with a mutation at codon 61 in *HRAS* compared to liver tumours in mice not exposed to arsenic, and suggests that this mutation might be associated with arsenic-induced oxidative stress [55]. Similarly, it is hypothesized that mutations in the tyrosine kinase domain of the epidermal growth factor receptor (EGFR) are responsible for the activation of the EGFR pathway [56], a common molecular feature of many cancers that is also observed in cell lines exposed to arsenic [57–61].

Oxidative stress can also lead to mutations and instability in mitochondrial DNA (mtDNA), which is associated with the development of skin cancers [62]. Mitochondria are involved in cell proliferation, cell death and abnormal cell differentiation, and therefore alterations in mtDNA structure and function have been correlated with carcinogenesis [63]. Additionally, ROS can also disturb the permeability of the mitochondrial membrane, leading to the aberrant expression of apoptosis related genes [64]. For that reason, As_2_O_3_ is used as a therapeutic agent, shown to induce apoptosis in leukemic cells [39].

#### Chemotherapeutic aspects of arsenic exposure

Interestingly, the carcinogenic and chemotherapeutic effects of arsenic might rely on common mechanisms [65]. In arsenic-induced carcinogenesis, the cells overcome the apoptotic effect that is observed after exposure to As_2_O_3_ through the activation of the nuclear factor erythroid-derived factor 2–related factor 2 (NRF2) pathway, responsible for the oxidative stress response, demonstrating that arsenic effects are both dose and time-dependent [66]. Taken together, cellular oxidative stress induced by arsenic exposure contributes to widespread genomic instability, which poses deleterious effects to the cell, and the individual [67, 68].

### Chromosomal alterations

Genomic instability resulting from cellular oxidative damage can also lead to further disruptions in chromosome structure and stability, including end-to-end fusion, abnormal sister chromatid separation, and aneuploidy [67]. Doses of arsenic around 10 μg/L have been shown to have an aneuploidogenic effect, illustrating the long-term risk of chronic low-dose exposure to arsenic [69]. Chromosomal aberrations of this sort are implicated in cancer development, possibly through the activation of proto-oncogenes [70]. Arsenic exposure may also disrupt microtubule assembly through interaction with the sulfhydryl groups of tubulin, leading to mitotic spindle complex malfunction [6, 71]. This can result in increased micronuclei formation, which is also associated with the onset of cancer [72, 73]. Another consequence of arsenic-induced genomic instability is the continued progression through the cell cycle despite DNA damage, accomplished through inhibition of the p53 mediated apoptotic response [74].

**Figure 4:**
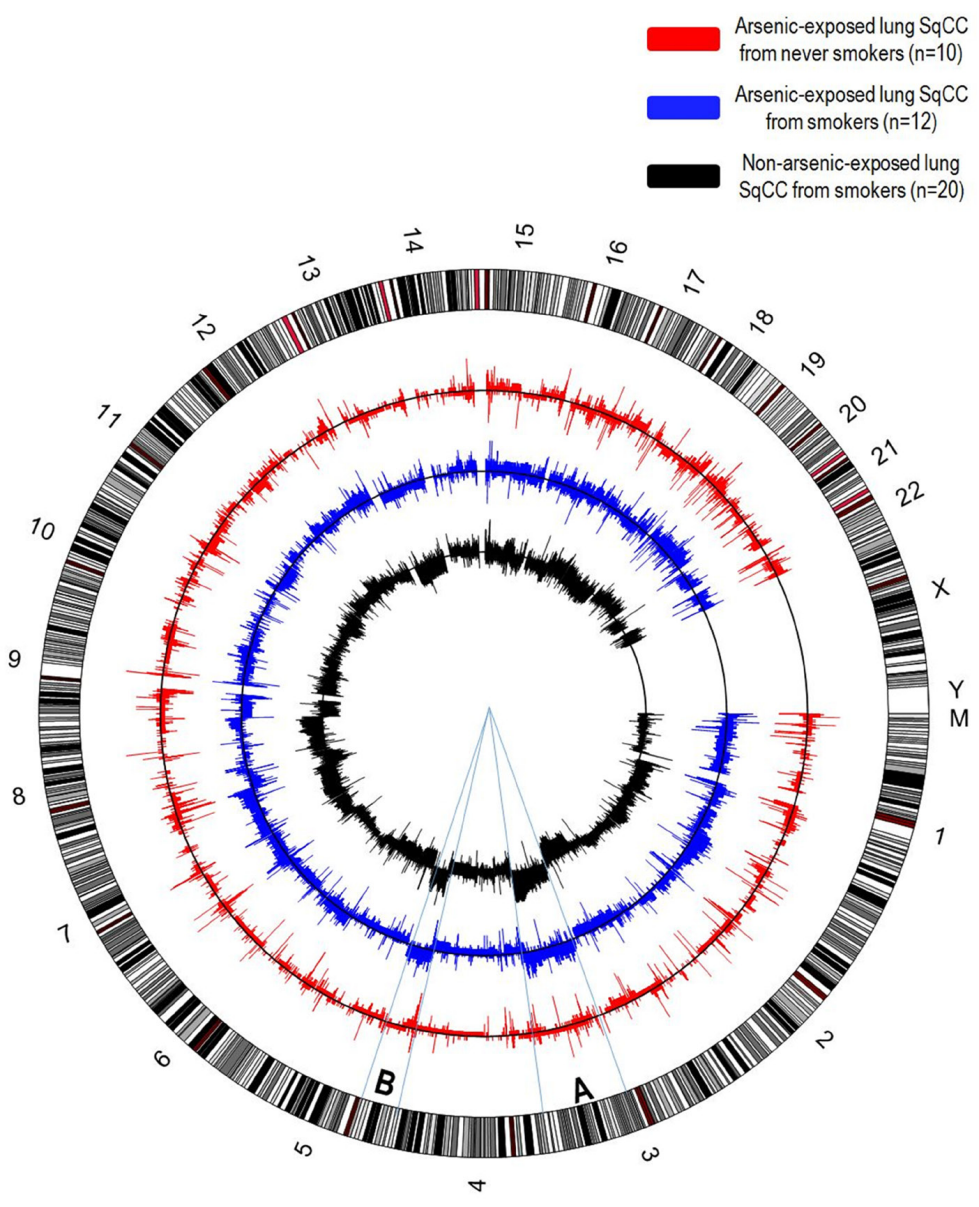
Circular representation of DNA copy-number alterations (CNAs) in lung squamous cell carcinomas Each chromosome of the human genome (hg19) is represented in the outer circle. Only lung squamous cell carcinomas were considered for this analysis, since this is the histological subtype more strongly associated with arsenic exposure. In arsenic exposed patients, there is an unusually high frequency of lung SqCC among never smokers, while this subtype is almost exclusively associated with smokers in non-arsenic related lung SqCC. CNAs detected in lung SqCCs arsenic-exposed, non-smoker patients (red, n=10), arsenic-exposed, smokers (blue, n=12) and non-arsenic exposed, smokers (dark grey, n=20) are shown. On each chart, the frequency of DNA gains among cases is shown above the black line indicating absence of alterations, while the frequency of DNA losses are shown below. Overall, the number of alterations observed in arsenic-exposed, non-smokers lung SqCCs are significantly lower than smokers. Interestingly, one of the most characteristic alterations described in lung SqCC (DNA gains 3q and 5p) exhibits a remarkable similarity among smokers, regardless of arsenic exposure status, while a low frequency of alterations is observed among non-smokers, arsenic-exposed patients (segments A and B).

In addition to chromosomal alterations and genomic instability, arsenic exposure is also related to DNA copy-number alterations (CNAs) (Figure 4), a key feature of tumour progression evidenced by the amplification of oncogenes and the deletion of tumour suppressor genes [75]. It has been demonstrated that lung squamous cell carcinoma exhibits both segmental DNA gains and losses after exposure to arsenic through dietary sources, compared to lung tumour genomes from smokers and non-smokers who have not been exposed to arsenic [76, 77]. Interestingly, this study implicated arsenic-induced DNA losses at the 9q12 locus, which is known to contain a gene from the *FOX*-gene family [76, 78]. *FOX*-gene family proteins are DNA-binding proteins that are involved with the regulation of transcription as well as DNA repair, some of which possess tumour suppressive functions while others display oncogenic features, and are frequently deleted or overexpressed through CNAs in many human cancers [79].

Conversely, it has been shown that in *CDKN1B* and *CDKN2A*-deleted cells, treatment with As_2_O_3_ resulted in increased signal patterns of these genes [80]. As *CDKN1B* and *CDKN2A* are members of a cell-cycle-inhibiting gene family, this suggests another possible mechanism of apoptotic induction by As_2_O_3_. Furthermore, CNAs may serve as prognostic factors for patients with APL, such as the deletion of the gene encoding CD56 by As_2_O_3_, which correlates with higher relapse-free survival [81]. Further characterization of chromosomal abnormalities and CNAs induced by arsenic will help to elucidate its carcinogenic mechanism and potentially implicate novel targets in therapeutic responses.

## EPIGENETIC FEATURES OF ARSENIC-INDUCED CANCER

During arsenic biotransformation, As^III^ is known to be methylated by S-adenosylmethionine methyltransferase (SAM) as part of the excretion process (Figure 2), which may lead to the depletion of SAM and consequent epigenetic disruption of the methylome [82–85]. This dependence of cellular detoxification and excretion of iAs on SAM and methyl group availability suggests that there may be epigenetic consequences of arsenic-exposure. Global DNA methylation levels and associated gene methylation changes play a critical role in cancer development, and also provide useful diagnostic and prognostic markers [86–88]. Differential DNA methylation patterns have been observed in individuals with high urinary arsenic concentrations, suggesting that these alterations may be important for non-genotoxic arsenic-induced carcinogenesis [89]. Arsenic exposure has been shown to induce global DNA hypomethylation, as well as specific gene promoter methylation changes through the alteration of CpG methylation status [90].

### Global hypomethylation

The methylation of arsenic is necessary for excretion, but this puts a high demand on the activity of several enzymes important in DNA methylation and epigenetic gene regulation, such as SAM and DNA methyltransferases (DNMTs). SAM is a cofactor that acts as a methyl-group donor for many biomolecules [91]. The production of methylated arsenic species leads to the depletion of SAM and a marked decrease in the availability of methyl groups in the cell [92]. Global hypomethylation can lead to chromatin remodeling, allowing for the transcription of previously inaccessible oncogenes and cancer-associated genes. It has been reported that exposure to 5 μM iAs over 29 weeks malignantly transformed cells, and was further correlated with an increased *S100P* and *HYAL1* expression, genes relevant to the malignant process [93]. This was accomplished through hypomethylation near the transcriptional start site of these genes. Evidence of global hypomethylation as a result of iAs exposure has been shown in multiple cancer types, including prostate, breast and liver cancers [36, 94, 95]. Furthermore, widespread DNA hypomethylation in hepatocytes is implicated in the increased expression of pro-growth genes, particularly estrogen receptor-α [95, 96]. Clinically, iAs exposure was observed to be a putative cause of significant DNA hypomethylation in adult peripheral blood mononuclear cells, suggesting possible involvement in lymphatic cancers [97]. Taken together, the current data suggest the significance of global DNA hypomethylation in arsenic-induced carcinogenesis.

### Promoter hypermethylation

Global methylation changes may be accentuated by specific promoter methylation alterations in cells exposed to chronic doses of iAs. In a genome-wide study, it was discovered that 2919 genes showed differential DNA methylation profiles when exposed to concentrations of iAs around current WHO guideline levels (at or above 10 μg/L), most of which were identified as CpG islands near the transcription start site [98]. Exposure to higher arsenic concentrations between 250-500 μg/L showed a similar relationship between iAs exposure and promoter hypermethylation [99]. Arsenic levels above 500 μg/L were associated with increased methyl acceptance capacity of promoter DNA, suggesting the onset of widespread hypomethylation at the point where the demand on the global methylation level is no longer sustainable [92, 99]. This displays the existence of a putative threshold at which global hypomethylation may become more prevalent in arsenic-induced carcinogenesis, which may have implications for early diagnosis and treatment of cancers associated with chronic exposure to iAs. These observations suggest that arsenic may be able to induce tumourigenesis and cancer progression through the epigenetic silencing of tumour suppressors as well as the epigenetic activation of oncogenes or associated genes. One of the most notable examples of this is the significant hypermethylation of the *TP53* promoter, the level of which was elevated in arsenic-induced skin cancers relative to skin cancers not resulting from arsenic exposure [100]. Evidence of promoter hypermethylation has been shown in a number of cancer types, including prostate, skin, and bladder, although the exact role of this in carcinogenesis has yet to be fully elucidated [101–103].

As_2_O_3_ treatment also displays a similar pattern as its therapeutic action may be through inhibition of DNMT expression level, global DNA hypomethylation or alternative epigenetic effects [104]. It can be suggested that this may reflect the carcinogenic mechanism of iAs exposure, but to an extent that leads to targeted cell death in APL cells. This was observed in prostate cancer cell lines, where DNA damage and hypomethylation triggered histone tail modification and chromatin remodeling, leading to the upregulation of pro-apoptotic genes [105]. In liver cells, treatment with As_2_O_3_ correlated with hypomethylation in the *cis*-regulatory sites of the promoter of *MYC* (a known cancer-associated gene), as well as hypermethylation in the promoter of *MAX* (a regulator of *MYC* and cell cycle) [106]. Thus, As_2_O_3_-based studies not only further the targeted therapy of cancer, but also help to elucidate the mechanism of arsenic toxicity, and in turn, its role in carcinogenesis.

## GENE EXPRESSION CHANGES

The numerous genomic and epigenetic changes resulting from iAs exposure culminate in the deregulation of a variety of genes. In Table 1, we summarize previously described coding-gene expression changes derived from arsenic exposure, demonstrating that this metalloid can alter crucial pathways involved in diverse cellular processes.

**Table 1:** Coding-gene expression changes linked to carcinogenesis resulting from exposure to arsenic

mRNA	Expression Change (non-exposed *vs*. exposed)	Arsenic Compound	Exposure Dose	Experimental model	Sample origin	Reference
DNA Repair and Stress Response						
ERCC1	Up	Drinking water	9.60–46.5μg/L in blood	Human sample	Frozen Peripheral Blood Lymphocytes	[110]
ERCC1	Down	NaAsO_2_	0.01–10μM	Cell line	Jurkat Lymphoblast Cells	[156]
POLB	Up	NaAsO_2_	2 or 50p.p.m.	Mice tissue	Female BALB/c Mice Lung tissue	[111]
POLB	Down	Drinking water	9.60–46.5μg/L in blood	Human sample	Frozen Peripheral Blood Lymphocytes	[110]
POLD2	Up	Drinking water	9.60–46.5μg/L in blood	Human sample	Frozen Peripheral Blood Lymphocytes	[110]
PARP1	Up	NaAsO2	2 or 50p.p.m.	Mice tissue	Female BALB/c Mice Lung tissue	[111]
PARP1	Down	MMA(III) or DMA(III)	0.1μM	Cell line	Human HeLa S3 Cells	[157]
APEX1	Up	NaAsO_2_	2 or 50p.p.m.	Mice tissue	Female BALB/c Mice Lung tissue	[111]
APEX1	Down	As_2_O_3_	0.005 – 5μM	Cell line	Normal Human Epidermal Keratinocytes (NHEK)	[158]
LIG1	Up	NaAsO_2_	2 or 50p.p.m.	Mice tissue	Female BALB/c Mice Lung tissue	[111]
OGG1	Up	NaAsO_2_	2 or 50p.p.m.	Mice tissue	Female BALB/c Mice Lung tissue	[111]
NQO1	Up	NaAsO_2_	2, 5 and 10μM	Cell line	Mouse hepa1c1c7 Cells	[159]
NQO1	Up	As^III^	0.005 – 5μM	Cell line	Normal Human Epidermal Keratinocytes (NHEK)	[158]
XPC	Down	As^III^	0.005 – 5μM	Cell line	Normal Human Epidermal Keratinocytes (NHEK)	[158]
XBP-1	Up	As_2_O_3_	5μM	Cell line	Murine Neuroblastoma Cells (Neuro-2a)	[160]
SESN1	Up	NaAsO_2_	5μM	Cell line	Human Breast Cancer Cell MCF-7 (p53+/+)	[161]
Cell Proliferation and Growth						
FOXM1	Up	As_2_O_3_	1μM	Cell line	Human Airway Epithelial Cell (NuLi-1)	[162]
GM-CSF	Up	NaAsO_2_	0 - 4μM	Cell line	Normal Human Epidermal Keratinocytes (NHEK)	[163]
PCNA	Up	NaAsO_2_	500nM	Cell line	Rat Liver Epithelial Cell (TRL1215)	[164]
CTBP1	Up	As_2_O_3_	1μM	Cell line	Normal Human Urothelial Cell (HUC1)	[165]
FOS	Up	As^III^	50μM	Cell line	Human HeLa S3 Cells	[166]
TGFB3	Up	NaAsO_2_	500nM	Cell line	Rat Liver Epithelial Cell (TRL1215)	[164]
Cell Death						
TNFRSF6	Up	NaAsO_2_	5μM	Cell line	Human Newborn Foreskin Cells (HFW)	[167]
FADD	Up	NaAsO_2_	5μM	Cell line	Human Newborn Foreskin Cells (HFW)	[167]
MCL1	Up	NaAsO_2_	5μM	Cell line	Human Newborn Foreskin Cells (HFW)	[167]
BAX	Up	As_2_O_3_	5μM	Cell line	Murine Neuroblastoma Cells (Neuro-2a)	[160]
BCL2	Down	As_2_O_3_	5μM	Cell line	Murine Neuroblastoma Cells (Neuro-2a)	[160]
Cell Cycle						
ATF3	Up	NaAsO_2_	5μM	Cell line	Human Breast Cancer Cell MCF-7 (p53+/+)	[161]
CDKN1A	Down	NaAsO_2_	0.1μM	Cell line	Human Keratinocyte Cell (HaCaT)	[112]
TP53	Up	As_2_O_3_	2μM	Cell line	Human Glioma Cells (U87MG and T98G)	[168]
MYC	Up	NaAsO_2_	500nM	Cell line	Rat Liver Epithelial Cell (TRL1215)	[164]
MYC	Up	NaAsO_2_	0 - 4μM	Cell line	Normal Human Epidermal Keratinocytes (NHEK)	[163]
RB1	Up	NaAsO_2_	500nM	Cell line	Rat Liver Epithelial Cell (TRL1215)	[164]
CDC6	Up	As_2_O_3_	1μM	Cell line	Human Airway Epithelial Cell (NuLi-1)	[162]
CDK2	Up	As_2_O_3_	1μM	Cell line	Human Airway Epithelial Cell (NuLi-1)	[162]
CDK1	Up	As_2_O_3_	1μM	Cell line	Human Airway Epithelial Cell (NuLi-1)	[162]
CDC25A	Up	As_2_O_3_	1μM	Cell line	Human Airway Epithelial Cell (NuLi-1)	[162]
CDC25A	Up	NaAsO_2_	5μM	Cell line	Human Newborn Foreskin Cells (HFW)	[167]
CCND1	Up	As_2_O_3_	1μM	Cell line	Human Airway Epithelial Cell (NuLi-1)	[162]
CCND1	Up	NaAsO_2_	5μM	Cell line	Human Bronchial Epithelial Cell (Beas-2B)	[56]
Cell Signaling						
EGFR	Up	As_2_O_3_	1μM	Cell line	Normal Human Urothelial Cell (HUC1)	[165]
TNFα	Up	NaAsO_2_	0 - 4μM	Cell line	Normal Human Epidermal Keratinocytes (NHEK)	[163]
TGFα	Up	NaAsO_2_	0 - 4μM	Cell line	Normal Human Epidermal Keratinocytes (NHEK)	[163]
H-Ras	Down	NaAsO_2_	50ppb	Mouse tissue	C57BL/6 Mice Offspring Hippocampal Nuclear Fractions	[164]
Raf-1	Down	NaAsO_2_	50ppb	Mouse tissue	C57BL/6 Mice Offspring Hippocampal Nuclear Fractions	[29]
VEGF	Up	NaAsO_2_	1 – 10μM	Cell line	Human Uroepithelial Cell (SV-HUC-1)	[169]
COX-2	Up	NaAsO_2_	1 – 10μM	Cell line	Human Uroepithelial Cell (SV-HUC-1)	[169]
HIF-1α	Up	NaAsO_2_	1 – 10μM	Cell line	Human Uroepithelial Cell (SV-HUC-1)	[169]
ERBB2	Up	NaAsO_2_	500nM	Cell line	Rat Liver Epithelial Cell (TRL1215)	[164]
ERBB2	Down	As_2_O_3_	1μM	Cell line	Normal Human Urothelial Cell (HUC1)	[165]
MAPK8	Up	AsIII	50μM	Cell line	Human HeLa S3 Cells	[115]
MAPK8	Up	NaAsO_2_	500nM	Cell line	Rat Liver Epithelial Cell (TRL1215)	[164]
H-RAS	Up	NaAsO_2_	500nM	Cell line	Rat Liver Epithelial Cell (TRL1215)	[164]
MET	Up	NaAsO_2_	500nM	Cell line	Rat Liver Epithelial Cell (TRL1215)	[164]

The disruption of multiple pathways can result in genomic instability and may lead to cancer development. A direct example is the alteration of the expression of genes involved with DNA repair. Arsenic exposure affects the ability of nucleotide excision repair (NER) in cell lines, which can enhance the mutagenicity of other carcinogens such as UV light [107, 108]. Among other factors, NER mechanisms are affected due to reduction of NER-associated genes (Table 1) [6, 109–112]. The poly-(ADP-ribose) polymerase 1 (PARP1) is a protein involved with DNA damage response that controls genomic stability and has been shown to be increased in arsenic exposed cell lines and mice samples [111–113]. Therefore, deregulation of PARP1 may be a possible mechanism of the induction of chromosomal instability and carcinogenesis [113].

Other characteristics of tumour cells that may be increased upon arsenic exposure are growth, proliferation and survival [14, 114]. For example, the PI3K/AKT pathway is affected by arsenic through the phosphorylation of AKT, activation of the JNK-STAT3 pathway and/or suppression of PTEN, an inhibitor of this pathway [6, 115–117]. Therefore, arsenic-induced activation of the PI3K/AKT pathway contributes to cellular transformation due to increased proliferation rates and induction of anchorage-independent growth [118].

The molecular damages caused by arsenic can be so extensive that cells are driven to undergo apoptosis [119]. This effect has been explored by the use of As_2_O_3_ as a chemotherapeutic for APL treatment [120]. The production of ROS reduces the mitochondrial membrane potential, leading to an increase in cytochrome-c release and consequent activation of caspases. Consequently, the normal protein ratio between the anti-apoptotic Bcl-2 and the pro-apoptotic Bax is also compromised, triggering apoptosis [64]. Studies show that As_2_O_3_ in high doses can induce apoptosis of B-cell leukemic cells, malignant lymphocytes, myeloma cells, and even cell lines derived from esophageal carcinoma and neuroblastoma [121–125]. However, in the case of arsenic-induced cancers, the cells can overcome the apoptotic effect derived from the DNA damage through the activation of factors involved in the antioxidant response, such as NRF2 [66].

## NON-CODING RNA EXPRESSION CHANGES

### MicroRNAs: Mediators of arsenic–induced carcinogenesis

The discovery that only a small portion of the transcribed human genome is translated into proteins led to a surge of interest in determining the role of non-coding RNAs (ncRNAs) in human diseases, especially regarding small ncRNAs [126–130]. There are three main classes of small ncRNAs: microRNAs (miRNAs), endogenous small interfering RNAs (endo-siRNAs) and PIWI-interacting RNAs (piRNAs) [131]. miRNAs are responsible for the post-transcriptional regulation of mRNAs and mainly repress translation through complementary binding along with RNA-induced silencing complex (RISC) assembly. These molecules have been extensively described and are known to be deregulated in cancer, playing important roles in cancer development and progression [132]. Correspondingly, arsenic studies associated with the deregulation of non-coding RNAs mainly describe alterations in miRNA expression, limiting our understanding of the association between arsenic exposure and the deregulation of long ncRNAs (lncRNAs) [133–135], or other types of small ncRNAs (Figure 3).

**Figure 5:**
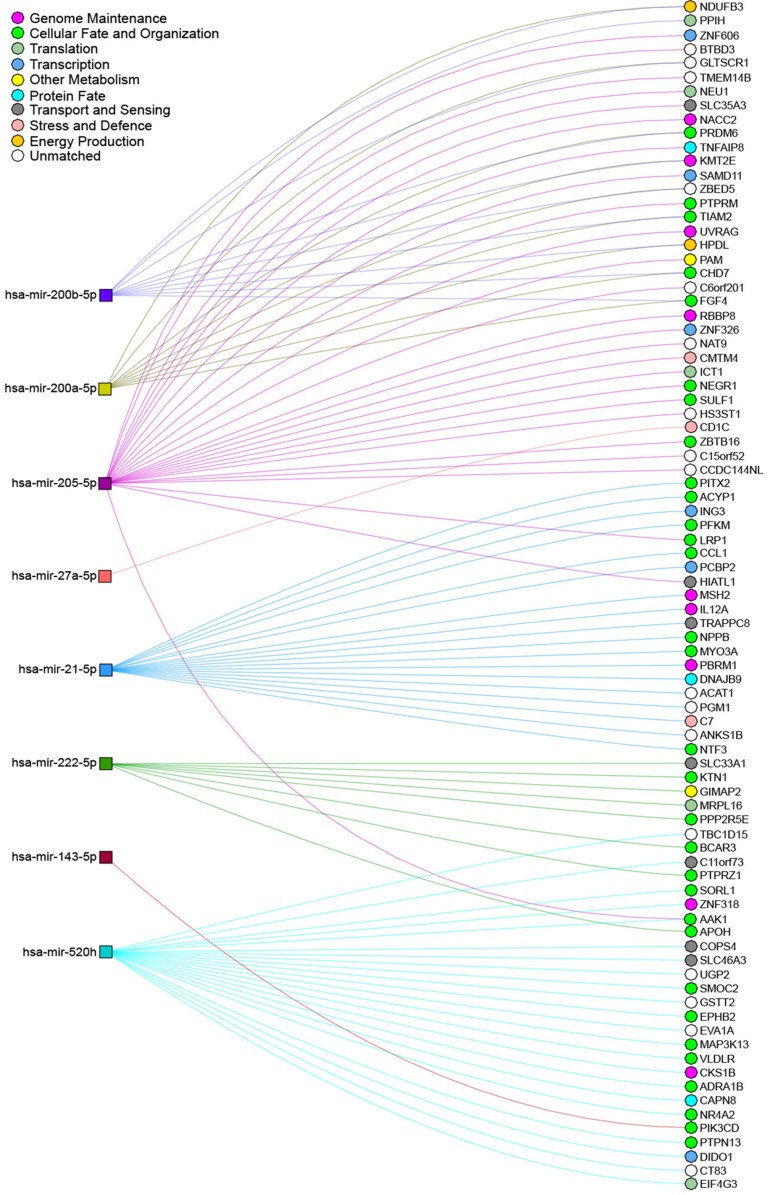
Network interactions between deregulated miRNAs and their predicted targets upon arsenic exposure miRNAs shown to be deregulated after exposure to arsenic and described in this review were inputted into miRDIP for gene target prediction, using the thresholds of the top 1% of mRNA transcripts predicted by at least 3 different prediction databases. NAViGaTOR [178] was used to visualize the interactions between these miRNAs and their predicted mRNA targets. miRNAs deregulated after exposure to arsenic are depicted by coloured square nodes, while their predicted mRNA targets are represented by circular nodes. Edges indicate predicted miRNA/mRNA interactions and are coloured according to the identity of the selected miRNA. The mRNA-target nodes are coloured as per to their association with Gene Ontology terms. Certain mRNAs appear to be shared by several of the miRNAs identified (i.e. FGF4, AAK1, CHD7, HPDL etc.), representing possible important cellular functions that are affected by arsenic exposure, such as cellular fate and energy production.

There is a strong link between arsenic exposure and the expression of miRNAs (Table 2), which may promote carcinogenesis. Many of these miRNAs are associated with cancer as they are responsible for negatively regulating oncogenes or tumour suppressors that are involved in several important cellular processes (Figure 5) [136]. The genes described in Table 2 and Figure 5 are only a representation of the known miRNAs that have differential expression when exposed to arsenic. In fact, one study showed 36 miRNAs to be consistently deregulated upon exposure to 2 μmol/L of sodium arsenite (NaAsO_2_) [137]. Of these, many are implicated in cancer. miR-150, for example, has been shown to be a circulating marker of prostate, colorectal, lung and pancreatic cancer [138–141]. In prostate cancer, miR-150 is upregulated, and is additionally correlated with tumour recurrence and metastasis, as well as poor overall survival [142].

**Table 2:** Selected miRNA expression changes resulting from exposure to iAs linked to important cellular processes

miRNA	Expression Change	Arsenic Exposure	Putative Target	Tissue / Cancer Type	Reference
miR-143	Down	5μM iAs	BCL2; BCL-XLApoptosis	Prostate cancer	[17]
miR-205	Down	1μM As_2_O_3_	AKT; mTORCell growth	Urothelial carcinoma	[165]
miR-27a	Down	Varied As_2_O_3_	Cell growth; apoptosis; migration	Breast cancer	[170]
miR-200b	Down	2.5μM NaAsO_2_	PKCα; Cell migration	Human bronchial epithelial cells; lung cancer	[171]
miR-21	Up	500μM NaAsO_2_	Cell proliferation promotion; apoptotic inhibition; acts on various tumour suppressors	Keratinocytes; Skin cancer (Melanoma); glioblastoma; prostate cancer	[172][173][174][175]
miR-200a	Up	500μM NaAsO_2_	Melanoma development	Keratinocytes; Skin cancer (Melanoma)	[172]
miR-520h	Down	Varied As_2_O_3_	PP2A/C (upregulation of this inhibits NF-κB); metastasis	Cervical cancer	[176]
miR-222	Up	1μM NaAsO_2_	ARID1A, PTEN; cell proliferation, migration	Lung cancer; Human lung epithelial BEAS-2B cells	[177]

Studies looking at the effects of As_2_O_3_ on non-coding gene expression show similar results. For example, miR-328 targets *hERG*, a gene encoding a subunit of a potassium ion channel. In the treatment of breast cancer, As_2_O_3_ is an effective therapy partly due to its action where it upregulates miR-328, thereby inducing apoptosis through the inhibition of *hERG* expression [143]. This highlights the importance of understanding the effects of arsenic exposure on more than the coding portion of the genome. miRNA-based studies have helped to uncover the details of the mechanism of arsenic induced carcinogenesis, which suggests that further characterization of other small non-coding RNAs involved in regulation may be of biological interest.

### PIWI-Interacting RNAs: Functions and prospective roles in arsenic-induced carcinogenesis

**Figure 6:**
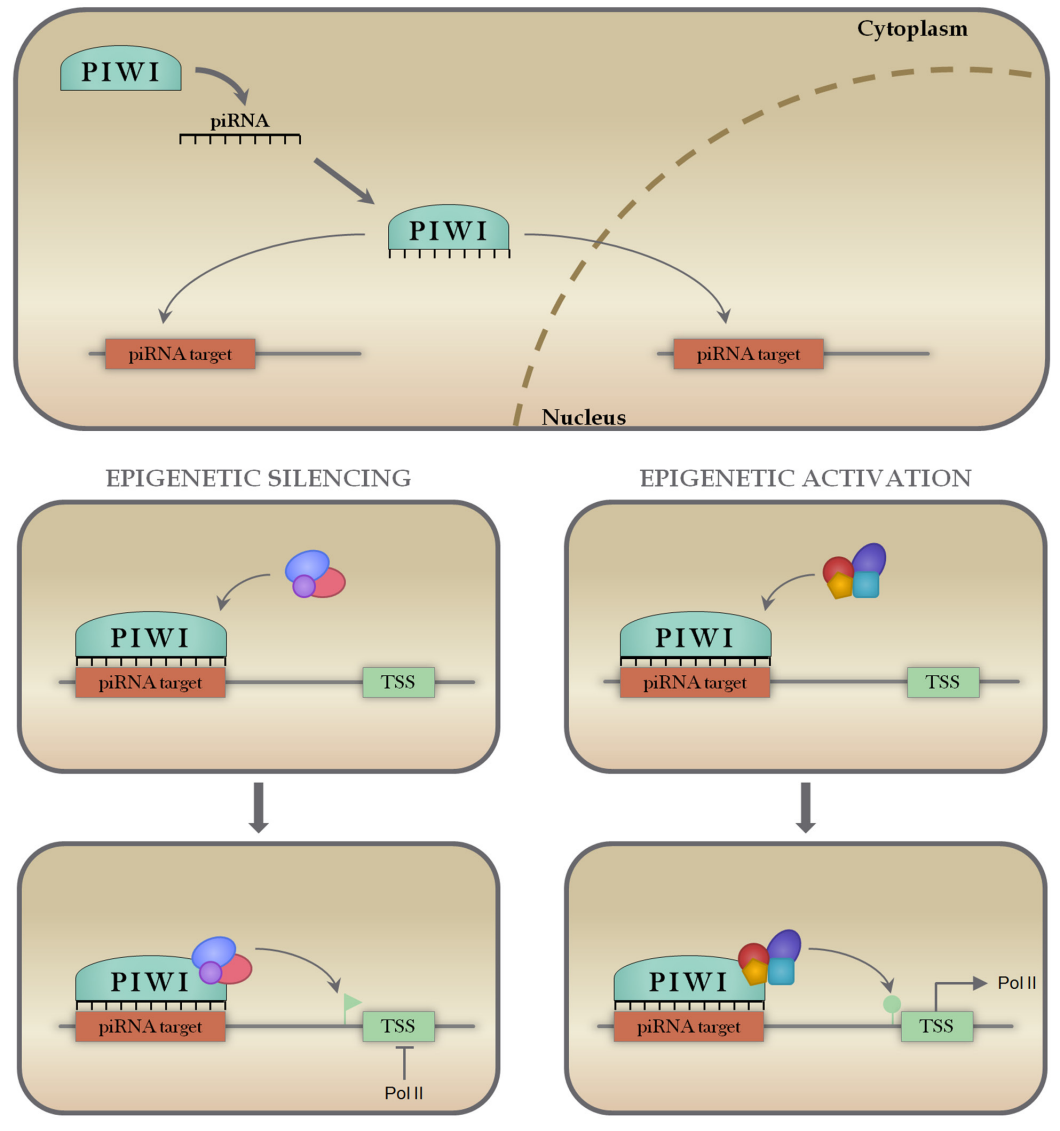
Mechanisms of piRNA action piRNAs associate with PIWI proteins in the cytoplasm, forming a ribonucleoprotein effector complex that is able to recognize and bind to complementary target sequences on DNA both in the cytoplasm and nucleus (panel A). When bound to the target sequence, piRNA-PIWI complexes can recruit epigenetic remodeling machinery (panels B and D) to either repress transcription through DNA methylation (panel C) or activate transcription through DNA acetylation or methylation removal (Panel E).

Although initially thought to be restricted to germ cells, piRNAs have been recently shown to be expressed in somatic tissues, displaying conserved mammalian biological functions [144, 145]. The uniqueness of this class is that they recognize complementary DNA sequences, instead of RNA sequences. Similarly to other classes of small non-coding RNAs, piRNAs are known to regulate gene expression through a small RNA-guided mechanism, in which piRNAs bind to the PIWI proteins of the Argonaute family forming the RISC, which can bind and regulate the expression of transcripts containing complementary sequences [146]. The main described function of piRNAs is the silencing of selfish genetic elements, mainly transposable elements (TEs), in the maintenance of genomic instability [147]. Later studies also demonstrated that piRNAs are able to promote epigenetic activation and even a miRNA-like transcript silencing [148–151]. In Figure 6 we illustrate these known functions, highlighting the importance of piRNAs as regulators of gene expression.

Since piRNAs are known to be involved with gene regulation and mainly with the control of genomic stability, it is likely that they are involved in a number of human diseases [126]. In fact, piRNAs display specific expression patterns that are markedly different across tissue types, between non-malignant and tumour tissues and even between different tumour subtypes [144]. As such, piRNAs have emerged as a highly promising area of study that might provide further knowledge on cancer biology and potentially improve tumour diagnosis and therapeutics.

As described here, the most well known and described mechanism of action of arsenic is the induction of oxidative DNA damage and disruption of permeability of the mitochondrial membrane. Numerous piRNAs were found to align with mitochondria specific small RNA sequences in cancer cells and also showed the coexistence of PIWI proteins and piRNAs in mitochondria [152]. Those findings suggest that the piRNA/PIWI complex might be involved in stress response and leads to the assumption that they might be important in arsenic-mediated tumourigenesis. Moreover, the piRNA/PIWI complex is known to be a major epigenetic regulator, being responsible for recruiting epigenetic machinery to binding sites, promoting epigenetic activation or silencing [148, 153]. Since epigenetic changes are another major mechanism for arsenic-induced cancer, this further supports the hypothesis that piRNAs may play important roles in arsenic-induced disease. Interestingly, so far there are no studies that have investigated the relation between piRNAs and arsenic-induced cancers. Therefore, this is an area that with further investigation, could improve our understanding on arsenic toxicology and therapeutics.

## CONCLUSION AND EMERGING QUESTIONS

Arsenic contamination of drinking water sources is a major problem worldwide. Clinical implications of the prevalence of arsenic groundwater contamination are evidenced by an impact on the incidence of cancer, even at low exposure levels [154]. This evidence suggests that the current guideline for maximum exposure to arsenic may still present a hazard to exposed populations. Limiting the effects of arsenic exposure on at-risk populations may require the implementation of strategies to manage groundwater concentrations, such as nanofiltration, adsorption and bioremediation [7, 155].

In this review article, we have discussed a spectrum of molecular aberrations induced by arsenic. Arsenic exposure is closely associated with DNA damage through the production of ROS, which may provide a distinct molecular signature. This type of oxidative damage can induce chromosomal instability including copy number alterations that lead to the amplification or deletion of certain loci, which has implications in carcinogenesis when an oncogene or tumour suppressor gene is involved. Arsenic exposure can also induce epigenetic changes, including global hypomethylation by the depletion of the global methyl pool, leading to aberrant gene expression, as well as alterations in promoter CpG island methylation status. Furthermore, arsenic exposure is associated with changes in both coding and non-coding gene expression, which not only affects critical-protein activity in cells, but also the regulation of coding-genes, through disruptions in miRNA and possibly other non-coding gene levels. Interestingly, the regulatory functions of piRNAs overlap with known mechanisms of arsenic toxicity and chemotherapeutic effects, leading to the assumption that piRNAs might play important roles in these mechanisms. However, our current understanding of the precise mechanism of arsenic-induced carcinogenesis is still far from comprehensive, and further work may look to characterize novel biological players involved.

The numerous health effects of arsenic ingestion demonstrate the complexity of the mechanisms linking arsenic exposure to disease. Arsenic has been shown to induce a number of damaging genomic and epigenetic effects, but the scope of these has yet to be determined. The study of these mechanisms will allow for a better understanding of both arsenic-induced cancer and arsenic-based therapies, which may lead to improved approaches for preventing exposure and reducing the onset of cancer, as well as the development of novel cancer therapeutics.
